# Splenic Heterogeneity in Focused Assessment With Sonography for Trauma (FAST) Scan Led to the Diagnosis of Grade 2 Splenic Injury in a Pediatric Blunt Abdominal Trauma

**DOI:** 10.7759/cureus.33128

**Published:** 2022-12-30

**Authors:** Nour F Alswaimil, Shahad A Alzahrani, Manal A Alfuraih, Dunya Alfaraj, Abdullah Alshahrani

**Affiliations:** 1 General Practice, King Fahad University Hospital, Khobar, SAU; 2 Emergency Department, Imam Abdulrahman Bin Faisal University, King Fahad University Hospital, Dammam, SAU; 3 Emergency Department, Imam Abdulrahman Bin Faisal University, Dammam, SAU

**Keywords:** trauma pediatric, computed tomography abdomen, focused assessment with sonography in trauma (fast), splenic trauma, blunt abdominal injury

## Abstract

Splenic injuries are one of the most common injuries following blunt abdominal trauma. It occurs in 32% of blunt abdominal trauma, with motor vehicle accidents being the most common cause. The patient may present with generalized abdominal pain or left upper quadrant pain associated with left shoulder pain. Hemodynamic instability is one of the most reliable signs of splenic injuries. A focused assessment with sonography for trauma (FAST) scan is the initial imaging used to assess for solid organ injury in the abdomen, followed by computed tomography (CT) scans. Evidence of free fluid in the abdomen in the FAST scan indicates a solid organ injury. However, the absence of the before-mentioned finding does not rule out the presence of solid organ injury.

Hereby a case of a 13-year-old Saudi male with left-sided abdominal pain after falling from a motorbike. A set of the investigation was done with insignificant results. However, FAST revealed a heterogenous appearance in the spleen. Thus, the patient subsequently underwent abdominal CT scans. The patient was managed conservatively and then discharged against medical advice (DAMA). Previously documented cases have mentioned the presence of free fluid in the abdomen in FAST scan in a stable patient leading to undergoing a CT scan and further managing trauma victim patients, which was absent in the present case.

## Introduction

Splenic injuries are the most common visceral injuries that could happen after blunt trauma to the abdomen [1,2]. Thirty-two percent of blunt abdominal trauma results in injuries to the spleen, most likely due to the high vascularity of the spleen and its anatomic location in the body [1]. The anatomic location of the spleen makes it vulnerable to injuries due to its proximity to the 9^th^, 10^th^, and 11^th^ rib in the left upper abdomen. Therefore, splenic injuries would be suspected in any blunt abdominal trauma involving the left lower part of the chest or left upper quadrant of the abdomen [2]. Motor vehicle accidents are the most frequently reported causes of splenic injuries [2,3]. Patients may present with left upper quadrant pain associated with left shoulder pain or generalized abdominal pain. Hemodynamic instability is one of the most reliable signs indicating splenic injuries [4].

Diagnosis and evaluation of splenic injury can be done by FAST, CT scan, and, less frequently, diagnostic peritoneal aspiration/lavage (DPA/DPL). Finding in the FAST scan suggesting splenic injury includes hypo-echoicrim around the spleen and/or free fluids in the abdomen. Grading of splenic injuries can be done using the American Association for the Surgery of Trauma (AAST) scale based on the injuries identified in the CT scan or during exploration [5]. Management of splenic injuries can be operative or non-operative. Guidelines have been markedly shifted towards non-operative management (NOM) to preserve the spleen’s physiological function [6]. NOM includes observation and angiographic embolization [5]. The decision on the modality of treatment will depend on the hemodynamic status of the patient and the radiological findings characterizing splenic injuries [6].

In this article, our discussion will revolve around the consideration of heterogeneity in solid organs, especially in the spleen, detected by ultrasound examination as an indication to proceed to CT scans even in low-grade or minor trauma.

## Case presentation

A 13-year-old Saudi male presented to our emergency department (ED) complaining of mild left-sided abdominal pain after falling from a motorbike on the left side of his body. The patient was not wearing a helmet nor elbow and knee protective pads and was riding at a low speed, and did not crash on any objects. There were no comorbidities nor prior surgical history noted. The patient was conscious, alert, and oriented with a temperature of 36.9 C (98.4 F), a pulse of 79/min, blood pressure of 114/72 mmHg, unlabored, and a regular respiratory rate of 18 breath/min, an oxygen saturation of 100% on room air, and a Glasgow Coma Scale (GCS) of 15 out of 15 at the time of presentation. Upon primary survey, the airway was intact and protected as he was able to phonate and handle his secretions with no neck pain or tenderness. On auscultation, there was equal bilateral air entry with equal chest rise and centralized trachea. The patient had a normal capillary refill time of fewer than 2 seconds with no long bone deformity and a stable pelvis. Pupils were examined and were round and reactive bilaterally. The patient neurological exam, including both motor and sensory, was unremarkable.

A chest X-ray was performed as an adjunct to the primary survey and was unremarkable for fractures, deviated trachea, pneumothorax, hemothorax, or subcutaneous emphysema. A FAST scan was done and revealed the absence of free fluid in both hepato-renal and splenorenal areas with no bladder injury or cardiac tamponade. However, a FAST exam revealed a disruption of the normal echogenicity texture of the spleen.

**Figure 1:**
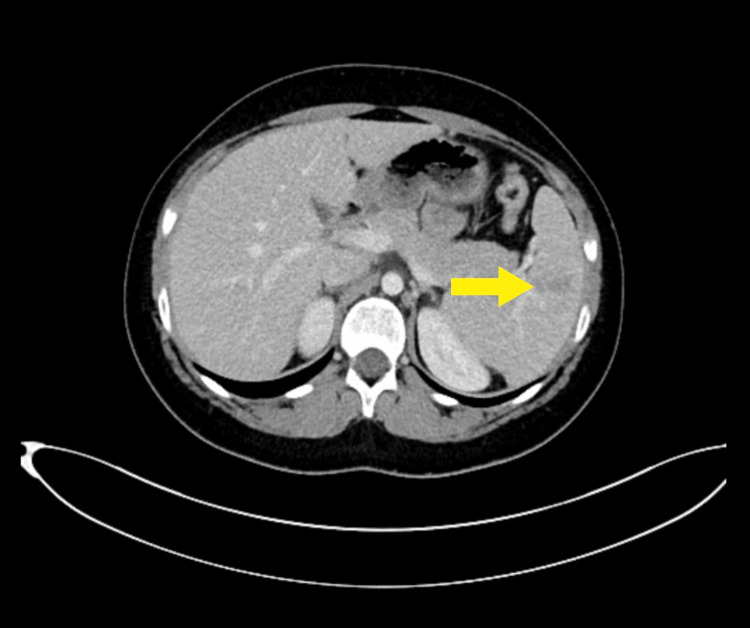
Axial view of contrast-enhanced computed tomography of abdomen suggesting grade 2 splenic lacerations showing multiple linear hypo-density extending from the splenic hilum reaching up to 2.5 cm.

**Figure 2:**
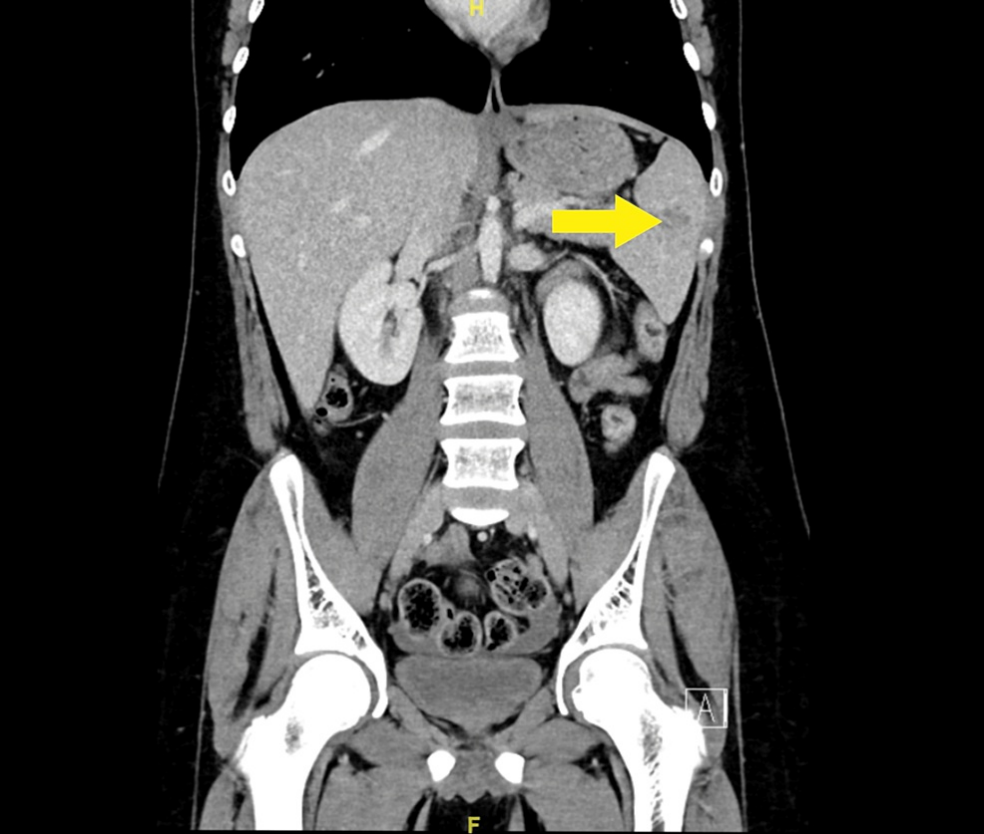
Coronal view of contrast-enhanced computed tomography of abdomen suggesting grade 2 splenic lacerations showing multiple linear hypo-density extending from the splenic hilum reaching up to 2.5 cm.

Routine biochemical markers, including complete blood count and renal and liver function tests, were normal, as well as arterial blood gas. CT abdomen demonstrated multiple linear hypodensities in the spleen from the splenic hilum reaching up to 2.5cm, suggestive of grade 2 splenic lacerations with no other trauma-related injury (Figures 1, 2).

A secondary survey of head-to-toe examination, with great attention to the abdomen, was unremarkable except for bilateral hand abrasions. The patient received 1 gram of paracetamol intravenously and was given 0.9NS 1 liter as a bolus, then maintained on D5 1/2NS 100ml/hr and shifted to the pediatric ward, and care did not continue because the patient’s mother signed DAMA. Before that, the risk of bleeding was explained, and instructions were given to the mother to return to the ED if the patient experienced abdominal pain, vomiting, or change in a bowel motion. Lastly, the patient was advised to bed rest for four weeks.

## Discussion

Blunt trauma can cause a range of injuries, from mild single-system injury to catastrophic multi-system damage [7]. The spleen is the most often injured solid organ in blunt abdominal trauma, followed by the liver, kidney, and pancreas [8]. Most splenic injuries present as hypovolemic shock and intraperitoneal bleeding immediately after trauma occurs. The diagnosis of splenic injury is often nonspecific based on physical examination and laboratory results [2]. FAST is the "first choice" diagnostic method since there are no particular biomarkers in the monitoring of spleen function in the early and posttraumatic phase [8]. It is well established that the FAST examination has advantages. It is non-invasive, easy to execute, and may be done while performing resuscitation. The technology is also portable and simple to reproduce if necessary. In most cases, it can be completed within 3 or 4 minutes. The test is effective in identifying intra-abdominal hemorrhage [7]. However, a negative FAST in pediatrics should not be falsely reassuring that intra-abdominal injury is absent [9]. In our case, the FAST scan was the initial test performed on the patient and showed no free fluid in the abdomen. However, a heterogeneous appearance was seen in the spleen.

Poor specificity for identifying the cause of hemoperitoneum and a limited capacity to identify solid organ damage (SOI) in the absence of free fluid have both been noted as limitations of ultrasonography in the context of blunt trauma. The usefulness of ultrasonography in this situation has been the subject of several pieces of research in the literature of emergency medicine, radiology, and surgery. The outcomes have been challenging to assess for the detection of SOI; for instance, one study reported sensitivities as low as 41% [10]. However, another study recorded sensitivities of higher than 90% [11]. The majority of researchers explain these disparities by pointing to the sonographer's experience, the injured organ being visualized, the extent of the injury, which criteria standard used to determine the presence of SOI, and the time ultrasound was being used relative to the timing of injury [12].

On the other hand, due to its speed, wide availability, diagnostic accuracy, and mostly noninvasive nature, contrast-enhanced CT scanning is currently the diagnostic imaging method of choice for the evaluation of hemodynamically stable patients with spleen damage [2]. Nonetheless, Moussavi et al. reported in their research that routine chest and abdominopelvic CT of conscious blunt trauma patients shorten hospitalization time but have little effect on patient outcome and can contribute to overtreatment of occult injuries. The use of a selective strategy should be investigated further to reduce radiation exposure and facility overuse [13]. In our case, based on the findings in the FAST examination suggestive of a splenic injury, a shared decision with the family has been made, and the patient underwent an abdominal CT scan which revealed a grade 2 splenic laceration.

The treatment method for splenic injuries are multifactorial depending on age, hemodynamic stability, injury grade, and comorbidity. Conservation (with or without angiography and embolization), spleen-preserving surgeries, and splenectomy are the current treatment options for splenic trauma patients. In the published literature, there is strong proof of the superiority of NOM over operative management of hemodynamically stable patients, as well as the increasing effectiveness of adjunctive therapies such as angiography with embolization, yet if NOM failed, the possibility of a second nonoperative reintervention is there; for instance, an attempt for splenic artery embolization after the failure of observation or proximal embolization after the failure of distal embolization [14]. The present case is in pediatric age with stable vital signs in whom preservation of the spleen is an essential priority.

NOM, on the other hand, poses a risk for delayed splenic rupture, rebleeding, and embolization-related complications, but the exact incidences of each were not reported in the searched literature. All in all, The study found a lack of universal standards for patient selection criteria and diagnostic and grading procedures available for NOM [14].

Djordjevic Et al. reported that it is challenging to anticipate the future course of complications based on the severity of the spleen injury. However, complications are more likely in high-grade injuries. Thus, all patients with NOM of spleen injuries should follow a follow-up ultrasonography protocol to detect any potentially fatal or serious complications [14]. Moreover, according to the evaluated literature, routine imaging follow-up CT scans of asymptomatic patients with lower-grade blunt splenic or hepatic injuries may not be suggested [15].

Therefore, in minor trauma patients with suspicious ultrasound findings, such as the presence of heterogeneity in the spleen, even in the absence of free fluid, one should consider an abdominal CT scan for further evaluation. In addition, following up with those patients in the US to detect potentially serious complications should be taken into consideration in clinical practice. However, additional research is required to define those patients population who will benefit from an ultrasound protocol follow-up post-trauma.

## Conclusions

Splenic injuries are the most common visceral injuries following blunt abdominal trauma. A FAST scan is the first tool used in trauma for assessing abdominal organ injuries by the presence of free fluid. However, the absence of the before-mentioned finding does not indicate the absence of solid organ injury. Hence, the presence of other findings, such as disruption of the organ echotexture, should permit further evaluation by a CT scan. Following discharge, an ultrasound follow-up protocol would benefit patients to detect serious complications in their early phase and will prompt early intervention. Nonetheless, to identify the patient population that will benefit from an ultrasound protocol follow-up post-trauma, further research is needed.
